# Factors influencing soft tissue profile changes following orthodontic treatment in patients with Class II Division 1 malocclusion

**DOI:** 10.1186/s40510-016-0125-1

**Published:** 2016-05-02

**Authors:** Suhatcha Maetevorakul, Smorntree Viteporn

**Affiliations:** Department of Orthodontics, Faculty of Dentistry, Chulalongkorn University, Henri Dunant Road, Pathumwan Bangkok, 10330 Thailand

**Keywords:** Class II Division 1 malocclusion, Factors, Orthodontic treatment, Soft tissue profile changes

## Abstract

**Background:**

Several studies have shown soft tissue profile changes after orthodontic treatment in Class II Division 1 patients. However, a few studies have described factors influencing the soft tissue changes. The purpose of this study was to investigate the factors influencing the soft tissue profile changes following orthodontic treatment in Class II Division 1 patients.

**Methods:**

The subjects comprised 104 Thai patients age 8–16 years who presented Class II Division 1 malocclusions and were treated with different orthodontic modalities comprising cervical headgear, Class II traction and extraction of the four first premolars. The profile changes were evaluated from the lateral cephalograms before and after treatment by means of the *X*-*Y* coordinate system. Significant soft tissue profile changes were evaluated by paired *t* test at a 0.05 significance level. The correlations among significant soft tissue changes and independent variables comprising treatment modality, age, sex, pretreatment skeletal, dental and soft tissue morphology were evaluated by stepwise multiple regression analysis at a 0.05 significance level.

**Results:**

The multiple regression analysis indicated that different treatment modalities, age, sex, pretreatment skeletal, dental and soft tissue morphology were related to the profile changes. The predictive power of these variables on the soft tissue profile changes ranged from 9.9 to 40.3 %.

**Conclusions:**

Prediction of the soft tissue profile changes following treatment of Class II Division 1 malocclusion from initial patient morphology, age, sex and types of treatment was complicated and required several variables to explain their variations. Upper lip change in horizontal direction could be found only at the stomion superius and was less predictable than those of the lower lip. Variations in upper lip retraction at the stomion superius were explained by types of treatment (*R*^2^ = 0.099), whereas protrusion of the lower lip at the labrale inferius was correlated with initial inclination of the lower incisor (L1 to NB), jaw relation (ANB angle), lower lip thickness and sex (*R*^2^ = 0.403). Prediction of chin protrusion at the soft tissue pogonion was also low predictable (*R*^2^ = 0.190) depending upon sex, age and initial mandibular plane angle (SN-GoGn). Additionally, age and sex also had mainly effect on change of the soft tissue profile in the vertical direction.

## Background

Facial esthetics is an important goal of treatment for contemporary orthodontics and it is one of the patient’s main reasons for seeking orthodontic treatment. The soft tissue of the face plays an important role in facial esthetics and the orthodontist is frequently questioned about facial changes after treatment. Thus, it is recognized by most orthodontists that success of orthodontic treatment is closely related to improvement of the soft tissue profile.

Class II Division 1 malocclusion is characterized by upper anterior teeth protrusion resulting in upper lip protrusion and convex facial profile, which are considered esthetically unfavorable. Treatment of this malocclusion comprises growth modification by orthopedic appliances such as headgear or functional appliances, orthodontic treatment with or without extraction in patients with mild to moderate skeletal discrepancies and orthognathic surgery in adult patients with severe skeletal discrepancies [1].

Several studies have shown soft tissue profile changes after orthodontic treatment in Class II Division 1 patients. Kirjavainen et al [2] found that after cervical headgear treatment, the nasolabial angle was increased and the interlabial gap was decreased, indicating upper lip retrusion, while the lip thickness and depth of the chin did not change. The upper and lower lips were retrusive after orthodontic treatment involving extraction of four premolars [3–5]. After using Class II intermaxillary elastics, the lower lip advanced relatively more than the upper lip and this contributed to an improvement of the lip relationship [6].

However, few studies have described factors influencing the soft tissue changes. Kasai [7] found that changes of the stomion and the lower lip could be predicted and strongly reflected the changes of the hard tissue, but changes in the upper lip showed a weaker correlation with the hard tissue change. Moreover, chin form was influenced by hard tissue structures such as the ANB angle and lower facial height, rather than that of incisor retraction. Oliver [8] found that patients with thin lips or a high lip strain displayed a significant correlation between incisor retraction and lip retraction, whereas patients with thick lips or low lip strain displayed no correlation. Moreover, soft tissue profile changes varied according to sex. The effect of extraction therapy on the facial profile was greater for a girl than for a boy [9].

Difference in treatment modalities is one of the factors influencing the profile change. In one study, the upper and lower lips were more retrusive in the extraction group than in the non-extraction group [4]. Janson et al [10] found that the profile changes following cervical headgear treatment or maxillary premolar extraction were similar.

Previous studies [7, 11, 12] were undertaken to scrutinize the factors influencing the soft tissue profile changes by means of correlation between the hard and soft tissue changes after treatment. The result indicated that prediction of the upper lip response from incisor position after treatment was low predictable. Therefore, the objective of this study was to investigate the following factors: pretreatment dento-skeletal and soft tissue morphology, age, sex and treatment modality that relate to the soft tissue changes. The results of the study should verify the soft tissue profile changes following orthodontic treatment in Class II Division 1 malocclusion and suggest the proper treatment modalities for the individual patient.

## Methods

The subjects comprised 50 boys and 54 girls age 8–16 years (mean age 11.6 ± 1.42 years) who received orthodontic treatment from 1988 to 2012 in a private clinic by the second author. This study was approved by the ethics committee of the Faculty of Dentistry, Chulalongkorn University.

### Inclusion criteria

1. Class II Division 1 malocclusion with molar Class II relationship and overjet larger than 5 mm.
2. No history of trauma that could affect facial growth and development.
3. Absence of congenital syndromes or defects, obvious facial asymmetry, extreme vertical disproportion, or congenitally missing teeth.
4. A complete orthodontic record indicating patient history, age, sex, type of treatment, and lateral cephalograms taken before treatment (T1) and after treatment (T2) from the same radiographic machine.

### Treatment protocols

Group I: Orthopedic treatment with cervical headgear followed by fixed appliances using the edgewise technique. The sample comprised 30 patients (15 boys, 15 girls) aged 8–13 years (mean age 10.9 ± 1.34 years). Each patient was in the mixed dentition stage with unerupted permanent maxillary second molars and with well-aligned lower teeth or mild crowding that could be corrected during the leveling phase. Skeletal analysis indicated skeletal Class II normal or deep bite malocclusion due to maxillary protrusion, with severe upper incisor protrusion. Facial profile should be improved when the mandible is moved forward. Patients with bimaxillary protrusion when the mandible is moved forward were excluded. The facial development evaluated from the hand wrist film had not passed the peak of pubertal growth. The patients were recommended to wear the cervical headgear that delivered 500 g per side via the permanent maxillary first molars for 12–14 h per day for distalization of the maxillary first molar so that Class I molar relation could be achieved, and there was adequate space for correction of the upper incisor protrusion without extraction. The fixed appliance edgewise technique was prescribed in the second stage to obtain Class I molar and canine relations with acceptable overbite and overjet.

Group II: Fixed appliances using edgewise technique, non-extraction with Class II traction. The sample comprised 30 patients (15 boys, 15 girls) aged 10–16 years old (mean age 12.1 ± 1.63 years). Each patient was in the permanent dentition stage with full eruption of the maxillary second molar, severe upper arch constriction and narrow intercanine width that inhibited forward movement of the mandible. Moreover, each patient had minor to moderate crowding that could be corrected simultaneously during arch expansion and leveling. The clinical examination indicated improvement of the soft tissue profile when the mandible moved forward to obtain Class I molar and canine relations. The fixed appliance edgewise technique was used for upper arch expansion, and Class II traction force 120–200 g per side was prescribed for full-time traction after obtaining arch compatibility.

Group III: Fixed appliance using the edgewise technique with extraction of the four first premolars. The sample comprised 44 patients (20 boys, 24 girls) aged 10–14 years old (mean age 11.7 ± 1.15 years). Each was in the permanent dentition stage, and cephalometric analysis indicated severe protrusion of the upper and lower incisors with less skeletal malocclusion indicating mainly a dentoalveolar problem.

At the end of treatment, all cases had Class I molar and canine relationships with a 2–3 mm overjet and an overbite was no more than one-third of the lower incisor crown height.

### Cephalometric analysis

**Fig. 1:**
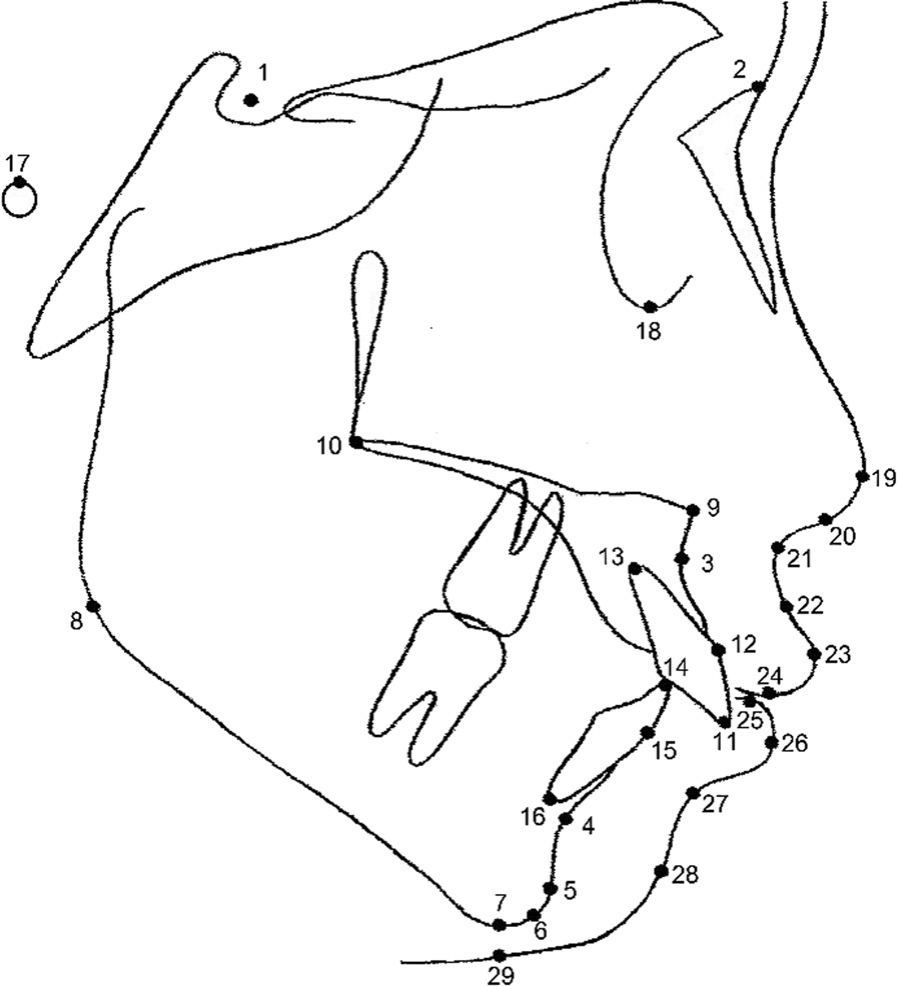
Cephalometric landmarks. *1* S (sella turcica), *2* N (nasion), *3* A (subspinale), *4* B (supramentale), *5* Pg (pogonion), *6* Gn (gnathion), *7* Me (menton), *8* Go (gonion), *9* ANS (anterior nasal spine), *10* PNS (posterior nasal spine), *11* maxillary central incisor edge, *12* the most anterior labial point of maxillary central incisor, *13* maxillary central incisor apex, *14* mandibular central incisor edge, *15* the most anterior labial point of mandibular central incisor, *16* mandibular central incisor apex, *17* Po (porion), *18* Or (orbitale), *19* Pr (pronasale), *20* Cm (columella), *21* Sn (subnasale), *22* Sls (superior labial sulcus), *23* Ls (labrale superius), *24* Ss (stomion superius), *25* Si (stomion inferius), *26* Li (labrale inferius), *27* Ils (inferior labial sulcus), *28* Pg’ (soft tissue pogonion), and *29* Me’ (soft tissue menton)

**Fig. 2:**
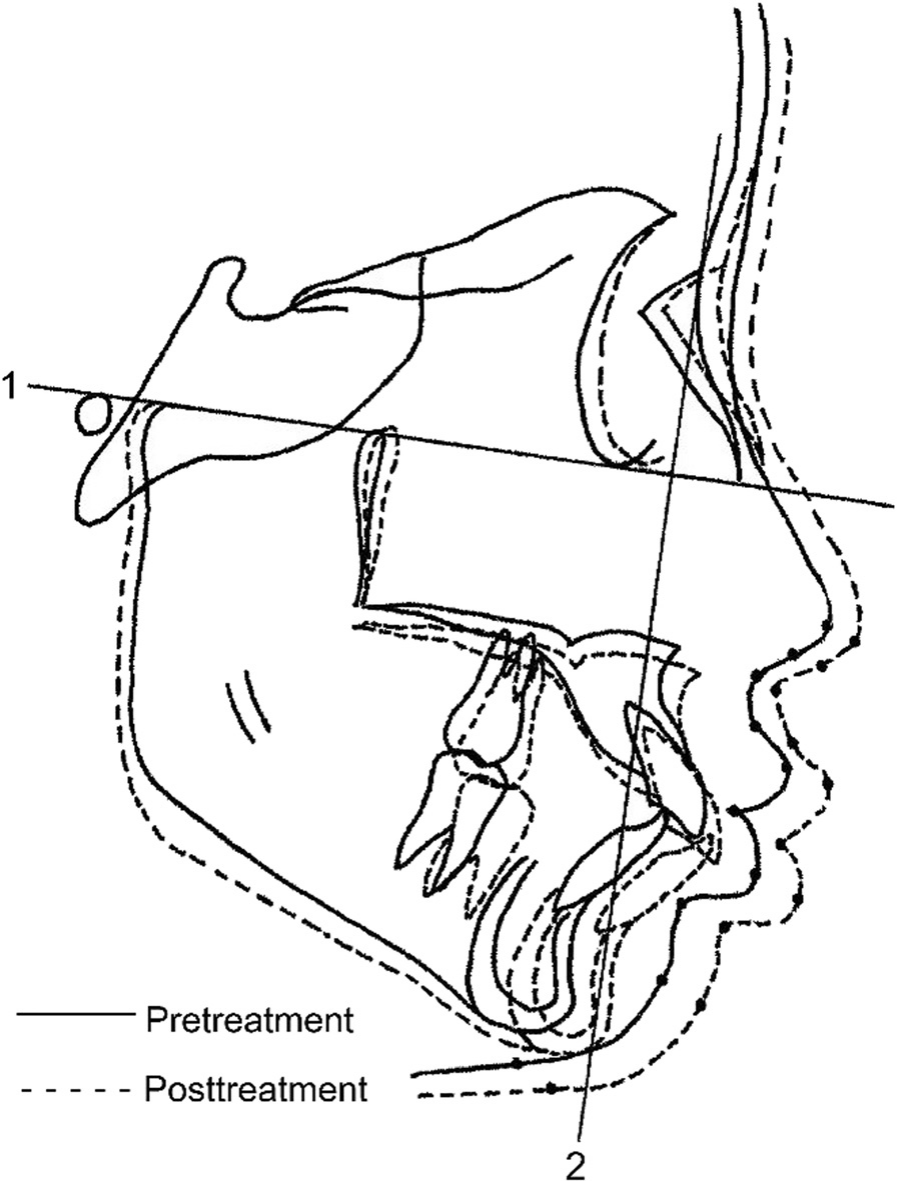
Reference points and reference planes utilized for evaluation of the soft tissue profile changes. *Line 1 X*-axis: FH plane of T1. *Line 2 Y*-axis: perpendicular plane to FH at the nasion point of T1

**Fig. 3:**
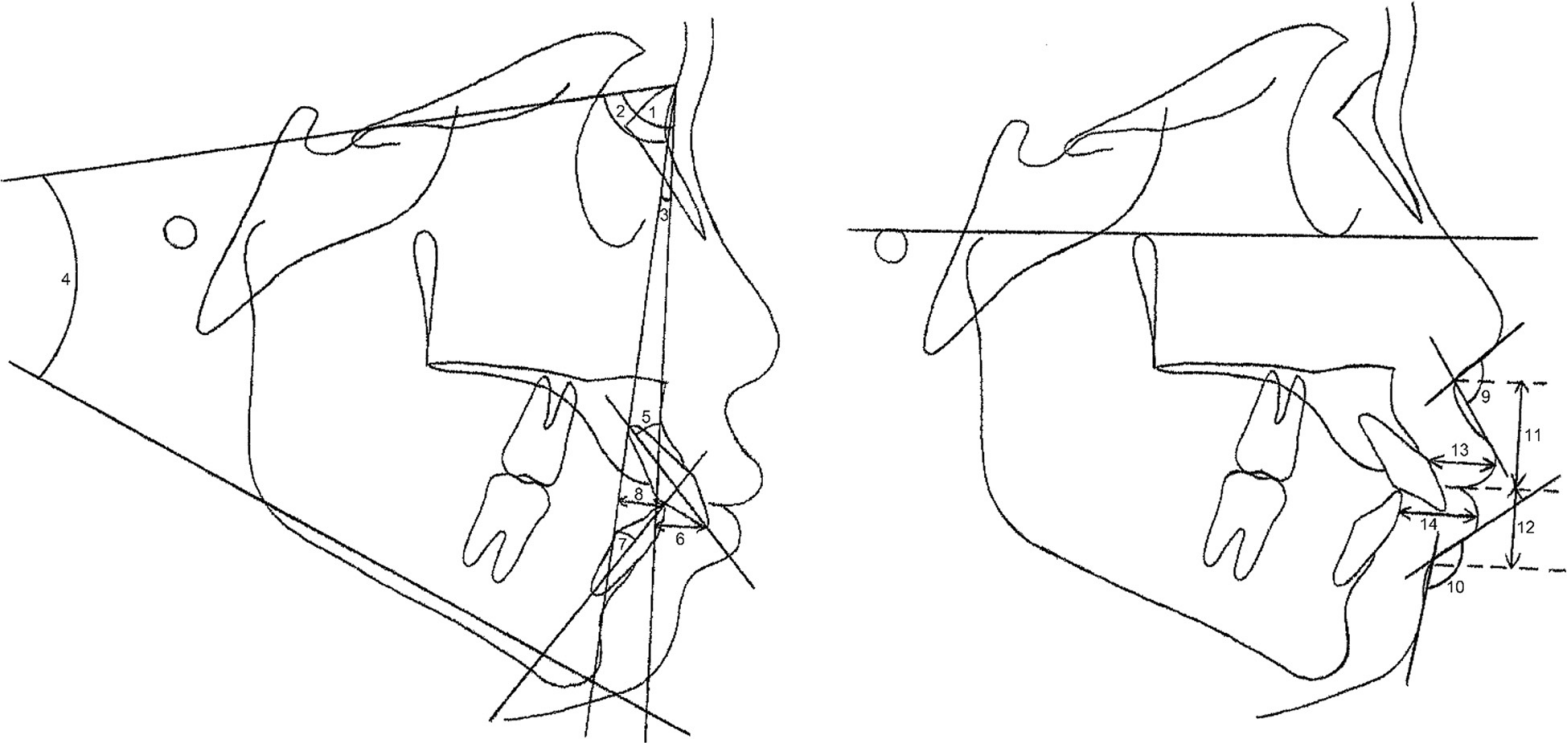
Angular and linear measurements utilized for evaluation of skeletal, dental, and soft tissue morphology before treatment. *1* SNA angle, *2* SNB angle, *3* ANB angle, *4* SN-GoGn angle, *5* U1-NA (angle), *6* U1-NA (linear), *7* L1-NB (angle), *8* L1-NB (linear), *9* nasolabial angle: NLA (Cm-Sn-Ls angle), *10* labiomental angle: LMA (Li-Ils-Pg’ angle), *11* upper lip length (Sn-Ss), *12* lower lip length (Ils-Si), *13* upper lip thickness (the labial surface of U1 to Ls), and *14* lower lip thickness (the labial surface of L1 to Li)

Both T1 and T2 films were traced by the same researcher on acetate paper, and the reference points representing hard and soft tissue structures were located (Fig. 1). Changes of the soft tissue profile were evaluated by means of the *X*-*Y* coordinate system where the Frankfort horizontal plane (FH) of the T1 film served as the *X*-axis and its perpendicular line at the nasion point served as the *Y*-axis (Fig. 2). The *X*-axis and *Y*-axis of the T1 film were transferred to the T2 film by structural superimposition on the stable structures of the anterior cranial base of the T1 film. The skeletal, dental, and soft tissue morphology before treatment were evaluated from the T1 film by means of linear and angular measurements (Fig. 3).

### Method error study

T1 and T2 films of 10 patients were randomly selected to retrace and remeasure all variables at least 2 weeks after the first measurement. The method error (ME) was estimated using Dahlberg’s formula [13]:

$$ \mathrm{ME}=\sqrt{\frac{{\displaystyle \sum {d}^2}}{2n}} $$

where *d* is the difference between the first and second measurements (millimeters or degrees) and *n* is the number of duplicated measurements.

### Statistical analysis

Significant soft tissue profile changes were evaluated by paired *t* test at a 0.05 significance level. For evaluation of the factors influencing soft tissue profile changes, correlations among significant soft tissue changes and independent variables comprising age, sex, treatment modality, pretreatment skeletal, dental and soft tissue morphology (Fig. 3) were evaluated by stepwise multiple regression analysis at a 0.05 significance level. The categorical variables comprising sex and treatment modalities were transformed to dummy variables with values 0 or 1.

## Results

The method errors of the variables evaluated by means of linear and angular measurements ranged from 0.22 to 0.69 mm and 0.35° to 2.64°, respectively. The method errors of the variables evaluated by means of the *X*-*Y* coordinate system ranged from 0.22 to 1.23 mm and 0.32 to 1.34 mm, respectively.

The soft tissue profile changes evaluated by means of the *X*-*Y* coordinate system of all subjects are presented in Table 1, indicating the significant changes of the soft tissue profile in horizontal and vertical directions, except for the horizontal positions of the Sls, Ls, and Si points (*p* ≤ 0.05). At the nasal area, all reference points exhibited significant forward and downward movements (*p* ≤ 0.05). At the upper lip area, the Ss point moved backward and downward, whereas the Sls and the Ls points only moved downward (*p* ≤ 0.05). At the lower lip area, the Li and Ils points moved forward and downward, whereas the Si point only moved downward (*p* ≤ 0.05). At the chin area, there were significantly forward and downward movements of the Pg’ and Me’ points (*p* ≤ 0.05).

**Table 1:** Changes of the soft tissue profile by means of the *X*-*Y* coordinate system

		All samples						
		(*n* = 104)						
Landmark (mm)		Pretreatment		Posttreatment		Changes		*t*
		Mean	s.d.	Mean	s.d.	Mean	s.d.	
Pronasale (Pr)	(*x*)	25.2	3.8	28.0	4.0	2.8	2.3	12.235**
	(*y*)	17.9	3.5	21.0	4.5	3.1	2.8	11.446**
Columella (Cm)	(*x*)	20.8	3.8	22.7	4.1	2.0	2.1	9.437**
	(*y*)	23.9	3.4	27.3	4.2	3.4	2.7	12.737**
Subnasale (Sn)	(*x*)	14.4	3.9	15.7	4.3	1.3	2.0	6.569**
	(*y*)	27.5	3.1	30.8	3.9	3.3	2.4	14.101**
Superior labial sulcus (Sls)	(*x*)	16.8	3.8	16.9	4.4	0.1	2.0	0.338
	(*y*)	35.2	3.3	38.8	4.1	3.7	2.7	13.982**
Labrale superius (Ls)	(*x*)	21.3	4.2	21.0	5.0	−0.3	2.2	−1.214
	(*y*)	42.5	3.8	46.5	4.5	4.0	3.2	12.715**
Stomion superius (Ss)	(*x*)	14.3	4.1	12.6	4.8	−1.6	2.4	−6.857**
	(*y*)	50.8	3.6	54.9	4.6	4.1	3.3	12.498**
Stomion inferius (Si)	(*x*)	12.3	5.2	12.2	5.0	−0.1	3.0	−0.439
	(*y*)	51.8	3.5	55.3	4.7	3.5	3.3	10.941**
Labrale inferius (Li)	(*x*)	17.1	5.6	17.7	5.4	0.6	3.0	2.019*
	(*y*)	58.5	3.8	63.0	4.9	4.5	3.4	13.294**
Inferior labial sulcus (Ils)	(*x*)	7.8	6.1	8.7	6.4	0.9	3.0	3.247**
	(*y*)	65.6	4.3	71.3	5.2	5.7	3.8	15.217**
Soft tissue pogonion (Pg’)	(*x*)	5.8	6.9	7.0	8.0	1.1	3.2	3.665**
	(*y*)	81.0	5.4	87.4	6.2	6.4	4.6	14.308**
Soft tissue menton (Me’)	(*x*)	−12.7	6.6	−11.7	8.1	1.0	3.0	3.277**
	(*y*)	95.8	5.5	102.9	6.7	7.1	4.1	17.786**

Significance **p ≤ 0.01; *p ≤ 0.05

The correlations among significant soft tissue changes and independent variables comprised of age, sex, treatment modalities, pretreatment dento-skeleton, and soft tissue morphology evaluated by the stepwise multiple regression analysis are presented in Table 2.

**Table 2:** Stepwise multiple regression models for soft tissue profile changes

Dependent variables (*Y*)	Prediction equation											*R* ^2^
	Constant (*β*_0_)	*β*_1_	*X*_1_	*β*_2_	*X*_2_	*β*_3_	*X*_3_	*β*_4_	*X*_4_	*β*_5_	*X*_5_	
Pr (*x*)	10.3	2.20	Sex	−0.55	Age							0.315
Pr (*y*)	22.9	−0.22	SNB	−0.64	Age	0.06	NLA	0.94	Sex			0.290
Cm (*x*)	7.33	1.98	Sex	−0.37	Age							0.264
Cm (*y*)	22.3	−0.80	Age	1.26	Sex	−0.18	SNB	0.05	NLA			0.352
Sn (*x*)	5.48	1.69	Sex	−0.29	Age							0.210
Sn (*y*)	19.1	−0.68	Age	1.26	Sex	−0.15	SNB	0.05	NLA			0.339
Sls (*y*)	25.9	−0.73	Age	1.61	Sex	−0.17	SNB					0.327
Ls (*y*)	31.6	−0.86	Age	2.24	Sex	−0.21	SNB					0.376
Ss (*x*)	−2.18	0.13	tx1	1.85	tx2							0.099
Ss (*y*)	32.8	2.40	Sex	−0.78	Age	−0.24	SNB					0.356
Si (*y*)	8.93	0.06	NLA	−0.24	SNB	0.44	Lower lip thickness					0.176
Li (*x*)	19.5	−0.21	L1-NB angular	0.58	ANB	−0.48	Lower lip thickness	1.78	Sex	−0.07	NLA	0.403
Li (*y*)	17.1	0.47	Lower lip thickness	−0.66	Age	−0.17	L1-NB angular	0.08	NLA	−0.18	SNB	0.272
Ils (*x*)	16.0	−0.06	LMA	1.85	Sex	−0.08	NLA	0.32	ANB			0.332
Ils (*y*)	30.8	2.31	Sex	−0.82	Age	−0.19	L1-NB angular	0.07	NLA	−0.19	SNB	0.319
Pg’ (*x*)	13.1	2.28	Sex	−0.17	SN-GoGn	−0.46	Age					0.190
Pg’ (*y*)	9.02	2.28	Sex	−0.86	Age	0.09	NLA					0.151
Me’ (*x*)	8.25	−0.22	SN-GoGn	1.78	Sex	−1.22	tx1	−1.88	tx2			0.204
Me’ (*y*)	22.3	−1.16	Age	3.27	Sex							0.309

*Y* = *β*_0_ + *β*_1_ *X*_1_ + *β*_2_ *X*_2_ + … + *β*_k_ *X*_k_

*β*_0_ = constant, *β*_1, 2,…, *k*_ = regression coefficient

*X*_1, 2,…, *k*_ = independent variables

Assumption for dummy variables in the equation

Sex: Boy = 1, Girl = 0

Treatment with headgear: tx1 = 1, tx2 = 0

Treatment with Class II traction: tx1 = 0, tx2 = 1

Treatment with extraction of four premolars: tx1 = 0, tx2 = 0

After treatment, forward movement of the nose at the Pr, Cm and Sn point was influenced by age and sex with 21.0–31.5 % predictive power. Meanwhile, downward movements of these points were correlated with not only age and sex, but also initial position of the mandible (SNB angle) and nasolabial angles with 29.0–35.2 % predictive power.

Backward movement of the upper lip studied at the Ss point was significantly influenced only by treatment modalities with 9.9 % predictive power. Downward movement at the Sls, Ls and Ss points was related with age, sex and pretreatment SNB angle with 32.7–37.6 % predictive power.

After treatment, the lower lip moved forward and downward. Forward movement studied from the Li point was explained by pretreatment lower incisor inclination, jaw relation (ANB angle), lower lip thickness, sex and pretreatment nasolabial angle with 40.3 % predictive power. Meanwhile, pretreatment labiomental angle, sex, pretreatment nasolabial angle and ANB angle had 33.2 % impact on forward movement of the Ils point. Predictive power of downward movement of the lower lip at the Si, Li and Ils points had high variations from 17.6 to 31.9 %. The least predictive power was found at the Si point (17.6 %) that was influenced by pretreatment nasolabial angle, SNB angle and lower lip thickness. The highest predictive value was found at the Ils point that was influenced by sex, age, pretreatment lower incisor inclination, nasolabial angle and SNB angle with 31.9 % predictive power.

At the chin area, forward movement of the Pg’ and Me’ points was influenced by the same variables: sex and mandibular plane angle. However, patient age involved only the Pg’ movement, meanwhile treatment modalities had impacted on the Me’ point. Vertical movement of the chin was better predicted at the Me’ point than the Pg’ point. Movement of the Me’ point could be explained by age, sex with greater predictive value (30.9 %) when compared with that of the Pg’ point (15.1 %) explained by sex, age and pretreatment nasolabial angle.

## Discussion

The soft tissue profile changes found in this study could be the result of treatment as well as facial growth, because all subjects were growing and there were no data of untreated Class II Division 1 malocclusion Thai subjects to differentiate between the effects of growth and treatment. The advantage of the study of profile change by means of the *X*-*Y* coordinate system is that this measurement can demonstrate the changes in horizontal and vertical directions separately.

The result indicated that not only different treatment modalities, but also other factors comprising age, sex, pretreatment dento-skeleton, and soft tissue morphology seemed to be related to the profile changes. Although several studies [12, 14–17] have described the relationship of the incisal movement to the profile changes, most emphasized the incisal position as well as the profile change after treatment. There was no previous scientific report about the relations of the initial patient morphology such as skeletal pattern, incisal position, and the soft tissue profile changes in terms of regression analysis. Therefore, multiple regression analysis was used in this study as a tool to investigate the influence of treatment modalities, the initial patient morphology, and other related factors on the soft tissue profile changes, since this information can be obtained before treatment and utilized for formulating the treatment plan. The predictive equation of the profile change based on initial patient morphology will enhance the decision regarding the best treatment modality.

The results manifested the negative correlation between pretreatment age and the vertical change of the soft tissue profile for all variables, which supported the results of Hodges et al [18]. Moreover, the study also showed the influence of sex on downward movement of all reference points, as presented in the prediction equations of the Table 2. Utilizing the prediction equation upon our assumption (boy (sex = 1) girl (sex = 0)), the calculation showed that boys had greater vertical changes than the girls due to more growth potential of the boys, thus supporting previous studies [19–22].

After treatment, the nose moved forward and downward due to facial growth, supporting the study of Hoffelder et al. [9], who concluded that the nose showed the greatest increase in height (8.65 mm) and length (13.7 mm) due to growth from 6 to 16 years. The regression analysis showed the correlation between sex, age, and the nasal growth, since the boys and the younger patients had greater change. Moreover, the vertical change of the two points was correlated with the SNB and nasolabial angles. There was more vertical change in patients with less SNB and greater nasolabial angles, indicating the vertical growth pattern of the face.

At the upper lip area, the regression analysis showed that the treatment modalities were the major factor influencing upper lip retrusion, evaluated from horizontal movement of the Ss point. None of the other variables produced a predictable regression. Change of the upper lip evaluated from our prediction equation indicated that the headgear treatment as well as extraction of four premolars had a similar effect on the upper lip retrusion. This result supported the study by Janson et al [10]. For the Class II traction group, there was little effect on the horizontal position of the upper lip. However, the predictive power of the treatment modalities on upper lip retrusion was low (9.9 %), and the result was in contrast with previous studies [12, 14–17] which concluded that the upper lip retraction was related to the upper incisor retraction, due to the difference of the independent variable between the initial position of the upper incisor utilized in this study and the change of the incisor position from the previous studies. For the vertical change in the upper lip, the regression analysis showed that the age, sex and the SNB angle played important roles on downward movement of the upper lip. For instance, the younger patients, the boys and the patients with less SNB angle had more vertical changes of the Sls, Ls and Ss points. These factors account for around 32.7–37.6 % of the predictive power of the three points.

At the lower lip area, the regression analysis showed that sex, the initial dento-skeletal, and soft tissue morphology were correlated with the horizontal change of the lower lip evaluated at the Li and Ils points. The patients with less ANB angle had less lower lip protrusion after treatment, which was consistent with the study of Zierhut et al [23]. Additionally, the patients with less lower incisor proclination before treatment seemed to have more lower lip protrusion after treatment. Moreover, patients with less nasolabial and labiomental angles had more lower lip protrusion after treatment. The thickness of the lower lip also played an important role in lower lip protrusion, which corresponded with the study of Oliver [8] who found a strong correlation between osseous and soft tissue changes in patients with thin lips. Moreover, the boys had more lower lip protrusion than that of the girls.

At the chin area, the regression analysis showed that horizontal changes of the chin at the Pg’ and Me’ points were different, as the treatment modalities had no effect on the horizontal change of the Pg’ point. For the Me’ point, the results indicated that not only the treatment modalities, but also sex and the mandibular plane angle were correlated with the horizontal change of the Me’. The boys and the younger patients had more forward movement of the chin. The patients with steeper mandibular plane angles indicating the vertical growth pattern of the face had less forward movement of the chin. Forward movement of the Me’ point calculated from the prediction equation (Table 2) was the least in the Class II traction group. This corresponded with the previous study of Ellen et al. [24] who concluded the effect of Class II traction on backward rotation of the mandible. Regarding the vertical change of the chin, sex and age played an important role on vertical change of the Pg’ and Me’ points. The boys and the younger patients had more downward movement of the chin. Moreover, the patients with a greater nasolabial angle also had more downward movement of the chin.

The multiple regression analysis provided the prediction equations of the soft tissue profile changes from the related dento-skeletal and other factors. These prediction equations have been tested upon the assumptions of the regression analysis that focused upon the nature of the error and the relations among the independent variables. Although the prediction equations of the soft tissue profile could be achieved, the feasibility of these equations should be considered as most of the predictive power of the independent variables was low (9.9–40.3 %) and required several independent variables to explain the profile changes, thus indicating that the nature of soft tissue profile changes were complicated and depended upon multiple factors. Lastly, the independent variables that only relied on initial characteristics of the patient might be inadequate.

Further study should be undertaken to test the relation between hard and soft tissue changes after treatment and compare the predictive power of this study with the further study, so that a suitable prediction equation will be obtained. Moreover, evaluation of soft tissue profile changes and the influencing factors in adult patients should be studied to eliminate the effect of growth.

## Conclusions

The soft tissue profile changes varied among different age, sex, treatment modalities, pretreatment skeletal, dental and soft tissue morphology. Prediction of the soft tissue profile changes following treatment of Class II Division 1 malocclusion from initial patient morphology, age, sex and types of treatment was complicated and required several variables to explain their variations. Upper lip change in the horizontal direction could be found only at the stomion superius and was less predictable than those of the lower lip. Variations in upper lip retraction at the stomion superius were explained by types of treatment (*R*^2^ = 0.099), whereas protrusion of the lower lip at the labrale inferius was correlated with initial inclination of the lower incisor (L1 to NB), ANB angle, lower lip thickness and sex (*R*^2^ = 0.403). Prediction of chin protrusion at the soft tissue pogonion was also low predictable (*R*^2^ = 0.190) depending upon sex, age and initial mandibular plane angle (SN-GoGn). Additionally, age and sex also had mainly an effect on change of the soft tissue profile in the vertical direction.
